# Comprehensive evaluation of dosimetric impact against position errors in accelerator‐based BNCT under different treatment parameter settings

**DOI:** 10.1002/mp.15823

**Published:** 2022-07-04

**Authors:** Ryo Kakino, Naonori Hu, Kayako Isohashi, Teruhito Aihara, Keiji Nihei, Koji Ono

**Affiliations:** ^1^ Kansai BNCT Medical Center, Osaka Medical and Pharmaceutical University Takatsuki‐shi Osaka Japan; ^2^ Institute for Integrated Radiation and Nuclear Science, Kyoto University Osaka Japan; ^3^ Department of Radiation Oncology Osaka Medical and Pharmaceutical University Takatsuki‐shi Osaka Japan

**Keywords:** boron neutron capture therapy, cyclotron‐based epithermal neutron source, Monte Carlo simulation, patient setup error

## Abstract

**Background:**

Patients who undergo accelerator‐based (AB) boron neutron capture therapy (BNCT) for head and neck cancer in the sitting position are generally uncomfortably immobilized, and patient motion during this treatment may be greater than that in other radiotherapy techniques. Furthermore, the treatment time of BNCT is relatively long (up to approximately 1 h), which increases the possibility of patient movement during treatment. As most BNCT irradiations are performed in a single fraction, the dosimetric error due to patient motion is of greater consequence and needs to be evaluated and accounted for. Several treatment parameters are required for BNCT dose calculation.

**Purpose:**

To investigate the dosimetric impacts (DIs) against position errors using a simple cylindrical phantom for an AB‐BNCT system under different treatment parameter settings.

**Methods:**

The treatment plans were created in RayStation and the dose calculation was performed using the NeuCure® dose engine. A cylindrical phantom (16 cm diameter × 20 cm height) made of soft tissue was modeled. Dummy tumors in the form of a 3‐cm‐diameter sphere were arranged at depths of 2.5 and 6.5 cm (denoted by *T*_2.5_ and *T*_6.5_, respectively). Reference plans were created by setting the following parameters: collimator size = 10, 12, or 15 cm in diameter, collimator‐to‐surface distance (CSD) = 4.0 or 8.0 cm, tumor‐to‐blood ratio (T/B ratio) using ^18^F‐fluoro‐borono‐phenylalanine = 2.5 or 5.0, and ^10^B concentration in blood = 20, 25, or 30 ppm. The prescribed dose was *D*_95%_ ≥ 20 Gy‐eq for both *T*_2.5_ and *T*_6.5_. Based on the reference plans, phantom‐shifted plans were created in 26 directions [all combinations of left–right (LR), anterior–posterior (AP), and superior–inferior (SI) directions) and three distances (1.0, 2.0, and 3.0 cm). The DIs were evaluated at *D*_80%_ of the tumors. The shift direction dependency of the DI in the LR, AP, and SI directions was evaluated by conducting a multiple regression analysis (MRA) and other analyses where required.

**Results:**

The coefficients of the MRA of the DIs for LR, AP, and SI shifts were −0.08, 2.16, and −0.04 (*p*‐values = 0.084, <0.01, and 0.334) for *T*_2.5_ and −0.05, 2.08, and 0.15 (*p*‐values = 0.526, <0.01, and 0.065) for *T*_6.5_, respectively. The analysis of variance showed that DIs due to the AP shift were significantly greater for smaller collimator sizes on *T*_2.5_ and smaller CSD on *T*_6.5_. Dose reduction due to SI or LR (lateral) shifts was significantly greater for smaller collimator sizes on both *T*_2.5_ and *T*_6.5_ and smaller CSD on T_2.5_, according to the Student's *t*‐test. There were no significant differences in the DIs against both the AP shift and the lateral shift between the different T/B ratios and ^10^B concentrations.

**Conclusion:**

The DIs were largely affected by the shift in the AP direction and were influenced by the different treatment parameters.

## 1 INTRODUCTION

Boron neutron capture therapy (BNCT) is conventionally performed using a nuclear reactor. However, owing to the difficulty of installing such equipment in hospitals, an alternative method that does not require a reactor is desired. Resultingly, accelerator‐based (AB) neutron source systems have been developed in many countries, and various types of accelerators, target materials, moderator systems, and irradiation systems have been considered.^1^, ^2^, ^3^, ^4^, ^5^, ^6^, ^7^

Unlike other modalities of radiation therapy, it is important to keep the distance between the collimator and the patient as short as possible to reduce the treatment time. The patient setting systems for BNCT installed in the Kansai BNCT Medical Center at the Osaka Medical and Pharmaceutical University are for lying or sitting positions, mainly used for the brain or head and neck regions, respectively. Patients who undergo BNCT for head and neck cancer in the sitting position are generally uncomfortably immobilized, and patient motion during this treatment may be greater than in other radiotherapies. Furthermore, the treatment time of BNCT is relatively long (up to approximately 1 h), which increases the possibility of patient movement during treatment. As most BNCT irradiations are performed in a single fraction, the dosimetric error due to patient motion cannot be ignored.

To accurately calculate the dose, several treatment parameters are required for BNCT dose calculation, such as the tumor‐to‐blood boron concentration ratio (T/B ratio) and blood ^10^B concentration.^8^ The T/B ratio is the ratio corresponding to the standardized uptake values (SUVs) calculated by positron emission tomography using ^18^F‐fluoro‐borono‐phenylalanine (FBPA‐PET); this is used to estimate the boron concentration in the tumor. The ^10^B concentration in blood is measured using inductively coupled plasma (ICP) from blood samples collected immediately before neutron irradiation, and the irradiation time is adjusted based on the obtained concentration. Other factors related to the irradiation field, such as the collimator size and collimator‐to‐surface distance (CSD), may affect the dose distribution. Three types of collimator sizes are available for clinical BNCT (circular field collimators with diameters of 10, 12, and 15 cm),^9^ and the size is selected based on the tumor size and location.

Lee et al. investigated the dosimetric impact (DI) against patient shift in reactor‐based BNCT through simulations using cylindrical and head phantoms.^10^ The authors quantitatively determined the DIs against shifts in the beam axis direction and superior–inferior, left–right directions for shallow and deep tumor locations. However, in their study, the treatment parameters set in the BNCT dose calculation were completely uniform; that is, the DIs for the characteristics of individual patients were not investigated. For example, they calculated the dose distribution by fixing the collimator size to a 14 cm diameter, and the tumor‐to‐normal tissue ratio to 3.5. They did not consider the CSD and the ^10^B concentration. Furthermore, as they used a reactor‐based neutron source, the tendency of DIs for a reactor system may be fundamentally different from that of an accelerator system. This is because the neutron energy spectra between the two systems are different,^1^ which results in different dose distribution in the human body.

Hence, we sought to investigate the DIs against position errors for an AB‐BNCT system installed at our institution (the Kansai BNCT Medical Center, Osaka Medical and Pharmaceutical University). We performed subgroup analyses of DIs by varying the treatment parameters. Because the BNCT settings significantly vary for each patient, a simple cylindrical phantom was used in the simulations to investigate the general characteristics of DIs.

## 2 MATERIALS AND METHODS

### 2.1 Treatment planning system

The treatment plans were created using the treatment planning system (TPS) RayStation version 9A (RaySearch Laboratories AB, Stockholm, Sweden) and the dose was calculated using the BNCT dose calculation program NeuCure® dose engine (Sumitomo Heavy Industries, Ltd., Japan). We have experimentally validated the dose engine system in our previous work.^9^ The dose engine utilizes a Monte Carlo simulation code PHITS version 3.2^11^ with nuclear data from the Japanese Evaluated Nuclear Data Library (version four) developed by the Japan Atomic Energy Agency.^12^

### 2.2 Phantom model

A cylindrical phantom (16 cm diameter × 20 cm height) similar to that used in the study of Lee et al.^10^ was modeled in the TPS. The phantom was made with homogeneous “Tissue soft” registered using RayStation. The mass density of the phantom was set to 1.000 g/cm^3^ and was composed of hydrogen, carbon, nitrogen, and oxygen with elemental weights of 0.101, 0.111, 0.026, and 0.762, respectively. Spherical tumors with a diameter of 3 cm were arranged at depths of 2.5 and 6.5 cm in the phantom and referred to as *T*_2.5_ and *T*_6.5_, respectively.

### 2.3 Reference and shifted plans

**FIGURE 1:**
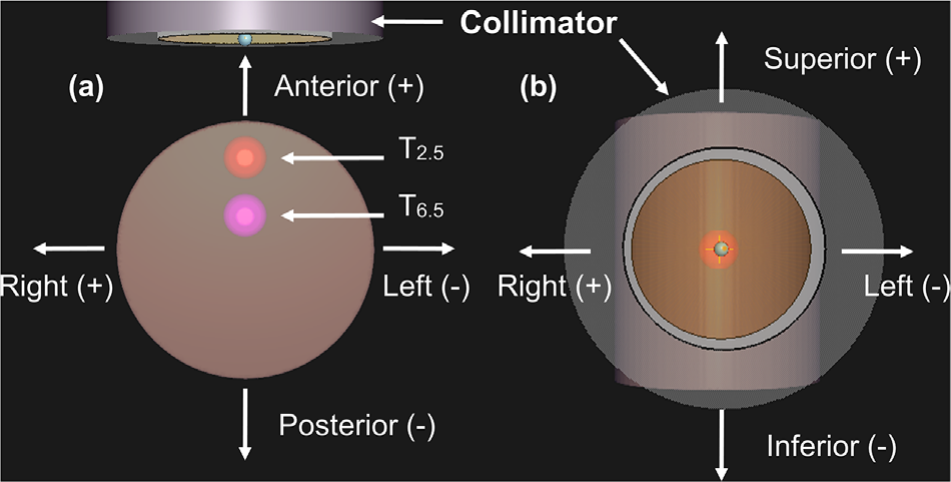
Layout of phantom shift directions in (a) axial view and (b) beam's eye view

Reference plans were created with the following parameters: collimator size = 10, 12, or 15 cm in diameter, CSD = 4.0 or 8.0 cm, T/B ratio = 2.5 or 5.0, and ^10^B concentration in the blood = 20, 25, or 30 ppm. Collimator sizes were selected by considering patients in certain clinical situations. When the tumor is close to the organs at risk, a smaller collimator size is selected to reduce the dose. The CSD is varied with the tumor location in patients. For example, patients with hypopharynx cancer produce larger CSD because the shoulder gets in the way. In our experience, a CSD of 4 cm is relatively close, whereas that of 8 cm is relatively far. The T/B ratio is unique for each patient. A T/B ratio of 2.5 or 5.0 is relatively small or large, respectively. The ^10^B concentration is also unique for each patient. A ^10^B concentration of 20, 25, or 30 ppm is relatively small, average, or large, respectively. The detailed calculation for relative biological effectiveness (RBE)‐weighted dose for BNCT is described in the Appendix. The prescribed dose for the tumor was the minimum dose that covered 95% of the tumor size (*D*_95%_) ≥ 20 Gy‐eq. The dose prescription that is commonly used in X‐ray therapy was applied because risk organs are not defined in the cylindrical phantom (although the dose is prescribed for risk organs in clinical BNCT.) From the reference plans, a total of 26 shift directions and three shift distances were considered.^13^ Figure 1 shows the shift directions of the phantom. The 3D shift ***s*** in (*LR*, *SI*, *AP*) can be expressed as follows:

(1) $$\begin{array}{ccc} s & = & \left\{\left(\mathrm{LR},\mathrm{AP},\mathrm{SI}\right)|\exists R,c>0,\mathrm{LR},\mathrm{AP},\mathrm{SI}\in \left\{0,\pm c\right\},\right.\\ & & \left.\sqrt{LR^{2}+AP^{2}+SI^{2}}\in \left\{1.0,2.0,3.0\right\}\right\}, \end{array}$$

where *c* is the shift distance in cm. Consequently, 78 ( = 26 × 3) shifted plans were calculated for each reference plan while keeping the irradiation time the same as in the reference plan. Here, the “−” sign represents left, posterior, or inferior, the “+” sign represents right, anterior, or superior, and the “0” sign represents no shift. For example, (−, 0, +) represents a shift in the left, no shift in AP, and a superior direction. The calculation grid size was set to 3.0 mm, and the statistical uncertainty in the Monte Carlo simulation was 10%. Additionally, the treatment parameters in the subgroup analyses were set as follows, unless stated otherwise: T/B ratio = 2.5, collimator size = 12.0, CSD = 4.0 cm, and ^10^B concentration in the blood = 25.0 ppm.

### 2.4 Analyses and statistics

In this study, DIs refer to the variation of the minimum dose covering 80% of the tumor size (*D*_80%_) (Gy‐eq).^14^ The shift distance dependency of the DIs was evaluated using the coefficient of variation (CV). CV was calculated using the following equation:

(2) $$CV=\frac{\sigma }{\mu },\;$$

where *σ* is the standard deviation of *D*_80%_ and *μ* is the mean of *D*_80%_ for all the shifted plans. The homogeneity index (HI)^15^ for comparing the dose‐volume histograms (DVHs) between *T*_2.5_ and *T*_6.5_ was calculated using the following equation:

(3) $$HI=\frac{D_{2\%}-D_{98\%}}{D_{p}},$$

where *D*_2%_ and *D*_98%_ are the highest and lowest 2% doses within the tumor size, respectively, and *D_p_* is the prescribed dose (≥ 20 Gy‐eq). HI represents the uniformity of the dose distribution inside the tumor size, and a lower HI value indicates a higher dose distribution uniformity. The minimum or maximum dose within the tumor size was not utilized because of the unreliability of the maximum or minimum dose at a given point in the Monte Carlo calculations.^16^

The shift direction dependency of the DI in the LR, AP, and SI directions was evaluated by conducting a multiple regression analysis (MRA). The coefficients and *p*‐values were calculated. The treatment parameter dependency of the DI in the AP direction was evaluated via a two‐way analysis of variance (ANOVA), which expresses whether the slopes of *D*_80%_ as a function of the AP shift are significantly different under the different parameters. The treatment parameter dependency of DI in the LR or SI (lateral) direction with a shift of 3 cm was evaluated through a Student's *t*‐test. The statistical significance was set at *p* < 0.05. All the statistical analyses were performed using Python (version 3.8.8) with the “scipy.stats” module.

## 3 RESULTS

### 3.1 Overall shifts

**FIGURE 2:**
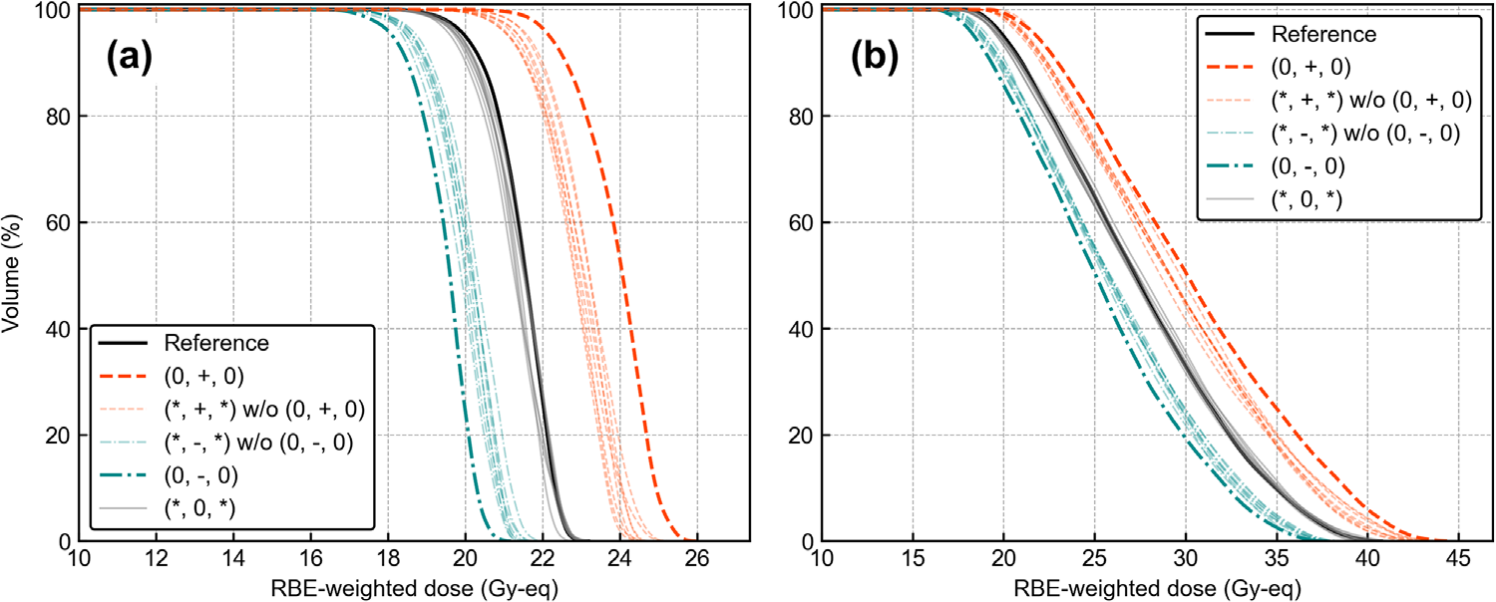
Dose‐volume histogram graphs with overall shift directions for | ***s*** | = 1.0 cm of (a) *T*_2.5_ and (b) *T*_6.5_

Figure 2 shows the DVHs of *T*_2.5_ and *T*_6.5_ for the overall shifts under | ***s*** | = 1.0 cm. The characteristics of the DVHs can be divided into different groups: (0, +, 0) group, (*, +, *) excluding (0, +, 0) group, (*, 0, *) excluding (0, 0, 0) (equal to reference plan), (0, −, 0), and (*, −, *) excluding (0, −, 0) group. The “*” sign represents a given direction. Table 1 lists the CV values for the different shift distances. The CV values arithmetically increased by 0.06 per 1 cm for *T*_2.5_ and by approximately 0.05 for *T*_6.5_.

**TABLE 1:** Coefficient of variation of overall shift plans with different shift distances and tumor depths

Shift distance (cm)	Coefficient of variations on *D*_80%_	
	*T*_2.5_	*T*_6.5_
1.0	0.06	0.05
2.0	0.12	0.10
3.0	0.18	0.16

**FIGURE 3:**
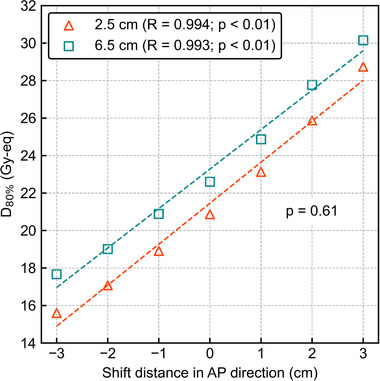
D_80%_ as a function of the shift distance in the AP direction. The *R* values in the legend represent the correlation coefficients. All data have a high correlation between the AP shift and *D*_80%_ and *p*‐values less than 0.01. Other *p*‐values in the graphs are evaluated by two‐way ANOVA

**FIGURE 4:**
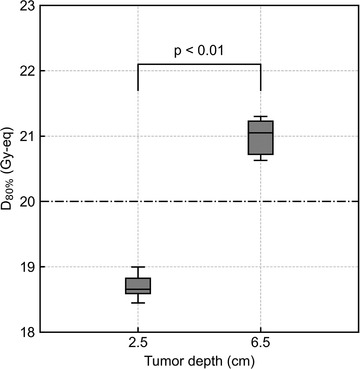
Comparison of *D*_80%_ as box plots for the lateral (superior–inferior and/or left–right) shift between *T*_2.5_ and *T*_6.5_. The *p*‐value was evaluated by the Student's *t*‐test

**FIGURE 5:**
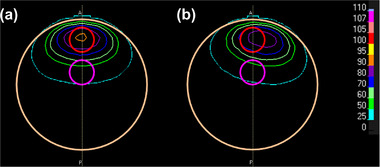
Boron physical dose of (a) no shift and (b) 3 cm shift for left direction. The color of the isodose curve indicates the relative values against the maximum dose inside the phantom

Table 2 presents the coefficients and their corresponding *p*‐values generated by the MRA of *T*_2.5_ and *T*_6.5_ for LR, AP, and SI shift directions. AP shift was only significant for the DIs (*p* < 0.01). Table 3 summarizes the HIs. The uniformity of dose distribution was much higher for *T*_2.5_ and slightly higher for smaller shifts. Figures 3 and 4 show a summary of the AP‐directional shift and lateral shift, respectively. The ANOVA result showed no significant difference in the DIs against the AP shift between different tumor depths (*p* = 0.61). The Student's *t*‐test showed significant differences in DIs between the different tumor depths (*p* < 0.01). Figure 5 shows an example of the difference in the boron dose distribution in the cylindrical phantom. The significant difference in boron dose distribution was visually observed in *T*_2.5_.

**TABLE 2:** Coefficients and *p*‐values in the multiple regression analyses of *T*_2.5_ and *T*_6.5_ for each shift direction

Shift direction	*T*_2.5_		*T*_6.5_	
	Coefficient ± std	*p*‐Value	Coefficient ± std	*p*‐Value
Left–right	−0.08 ± 0.05	0.084	−0.05 ± 0.08	0.526
Anterior–posterior	2.16 ± 0.05	< 0.01	2.08 ± 0.08	< 0.01
Superior–inferior	−0.04 ± 0.05	0.334	0.15 ± 0.08	0.065

Abbreviation: std, standard deviation.

**TABLE 3:** Homogeneity indices of *T*_2.5_ and *T*_6.5_ for three different shift distances

Shift distance (cm)	*T*_2.5_	*T*_6.5_
1.0	0.16 (0.14–0.18)	0.41 (0.36–0.47)
2.0	0.18 (0.14–0.20)	0.46 (0.35–0.52)
3.0	0.20 (0.13–0.24)	0.51 (0.32–0.60)

### 3.2 Effect of collimator size

**FIGURE 6:**
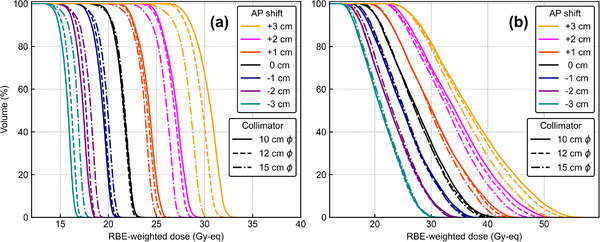
Dose‐volume histograms for different shift distances in the anterior–posterior direction and different collimator sizes for (a) *T*_2.5_ and (b) *T*_6.5_. AP, anterior–posterior; RBE, relative biological effectiveness

**FIGURE 7:**
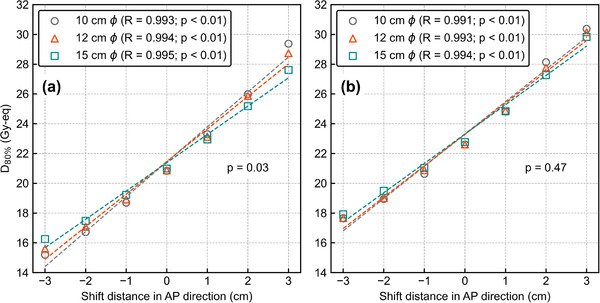
*D*_80%_ as a function of the shift distance in the anterior–posterior direction with different collimator sizes for (a) *T*_2.5_ and (b) *T*_6.5_. The *R* values in the legend represent the correlation coefficients. All data have a high correlation between the anterior–posterior shift and *D*_80%_ and *p*‐values less than 0.01. Other *p*‐values in the graphs are evaluated by two‐way ANOVA

Figure 6 shows the DVHs of the reference and AP‐shifted plans under three collimator sizes. Greater DIs were observed for smaller collimator sizes. Figure 7 shows the *D*_80%_ dose as a function of the AP‐shift distance with different collimator sizes. The ANOVA result showed a significant difference in these DIs against the AP shift between the three collimator sizes for *T*_2.5_ (*p* = 0.03) but no significant difference for *T*_6.5_ (*p* = 0.47).

**FIGURE 8:**
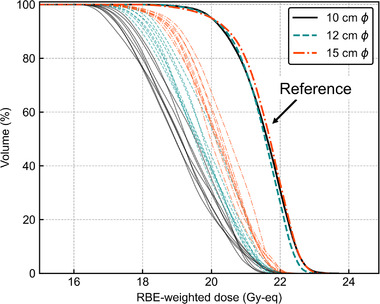
Dose‐volume histograms of the lateral (superior–inferior and/or left–right) shift direction by 3 cm under different collimator sizes for *T*_2.5_. Thick lines represent reference plans and thin lines represent shifted plans

**FIGURE 9:**
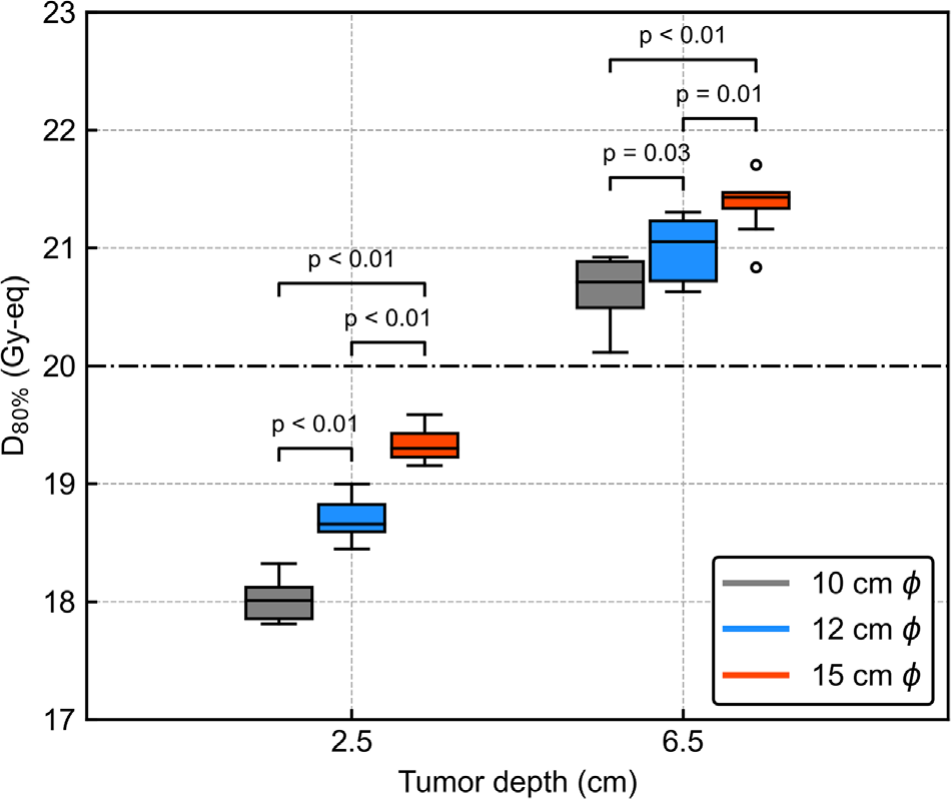
Box plots of *D*_80%_ for the 3 cm lateral shift with different collimator sizes. *P*‐values in the graphs are evaluated by the Student's *t*‐test

Figure 8 shows the DVHs of the reference and 3‐cm lateral‐shifted plans for the three collimator sizes for *T*_2.5_. A greater dose reduction on *D*_80%_ was observed for smaller collimator sizes. Those for *T*_6.5_ are summarized in Figure S3 in the Supporting Information. Figure 9 shows the box plots of *D*_80%_ for the 3 cm lateral shift with different collimator sizes. The Student's *t*‐test showed significant differences in DIs between all the collimator sizes (*p* < 0.05). The normalized percentage depth thermal neutron flux and off‐axis ratio referred by Hu et al. are also summarized in Figures S1–2.^9^ The DIs variations due to the different collimator sizes were agreed with these profiles.

### 3.3 Effect of collimator‐to‐surface distance

**FIGURE 10:**
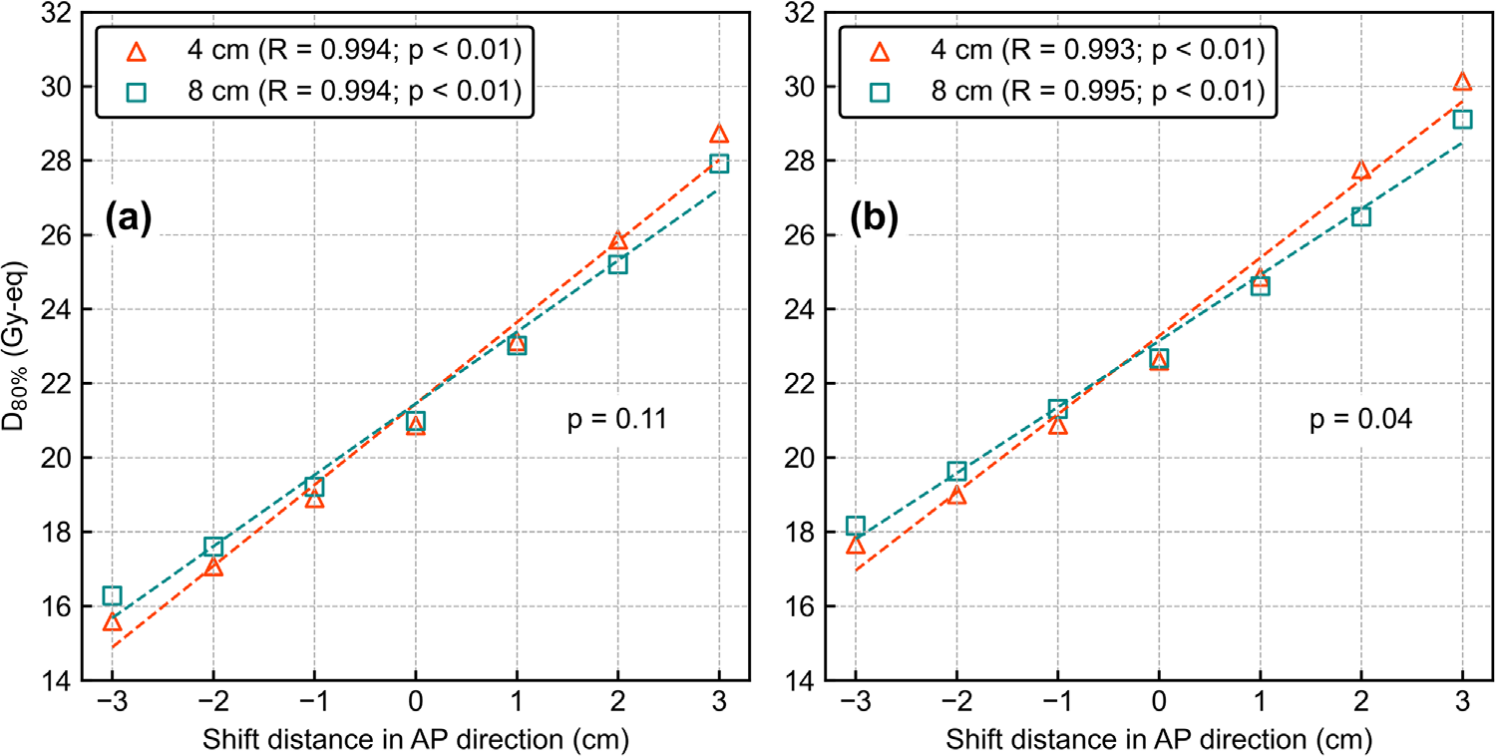
*D*_80%_ as a function of the shift distance in the AP direction with different collimator‐to‐surface distances for (a) *T*_2.5_, and (b) *T*_6.5_. The *R* values in the legend represent the correlation coefficients. All data have a high correlation between the anterior–posterior shift and *D*_80%_ and *p*‐values less than 0.01. Other *p*‐values in the graphs are evaluated by two‐way ANOVA

**FIGURE 11:**
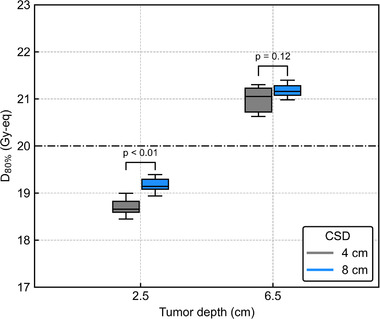
Box plots of *D*_80%_ for the 3 cm lateral shift with different collimator‐to‐surface distances. *P*‐values in the graphs are evaluated by the Student's *t*‐test

Figure 10 shows *D*_80%_ as a function of the AP‐shift distance with different CSDs. The ANOVA results showed a significant difference in the DIs against the AP shift between the CSDs for *T*_6.5_ (*p* = 0.04). There was no significant difference for *T*_2.5_ (*p* = 0.11). Figure 11 shows the box plots of *D*_80%_ for the 3‐cm lateral shift with different collimator sizes. The Student's *t*‐test showed significant differences in DIs between the various CSDs for T_2.5_ (*p* < 0.01). There was no significant difference for *T*_6.5_ (*p* = 0.12). The DVHs of the AP‐shifted and lateral‐shifted plans with different CSDs are also summarized in Figures S4–5. Figure S6 shows the boron dose falloff as a function of the CSD. The slope of the boron dose falloff tapers off at deeper locations, and this tendency agreed with the results that the DIs are smaller for larger CSD.

### 3.4 Effect of T/B ratio

**FIGURE 12:**
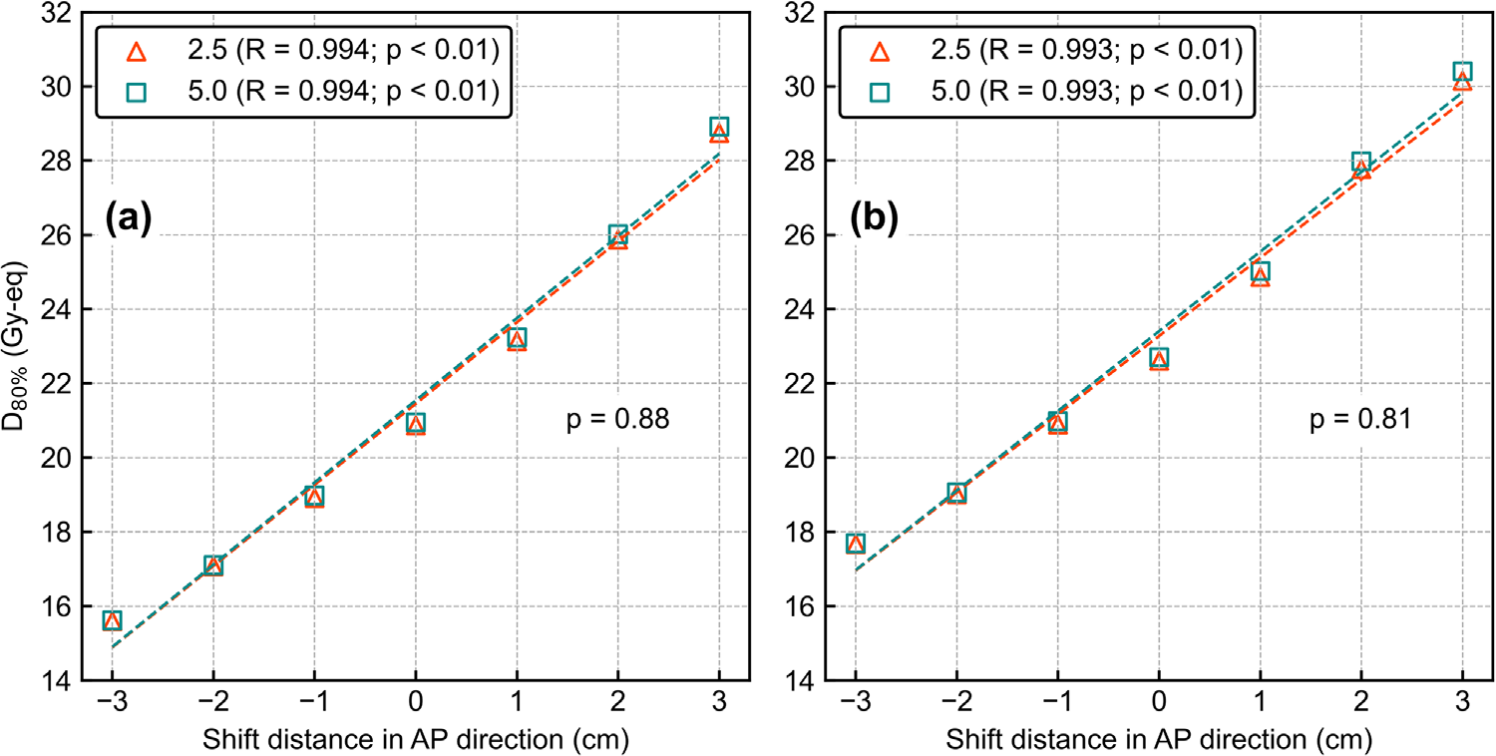
*D*_80%_ as a function of the shift distance in the AP direction with different T/B ratios for (a) *T*_2.5_ and (b) *T*_6.5_. The *R* values in the legend represent the correlation coefficients. All data have a high correlation between the anterior–posterior shift and *D*_80%_ and *p*‐values less than 0.01. Other *p*‐values in the graphs are evaluated by two‐way ANOVA

**FIGURE 13:**
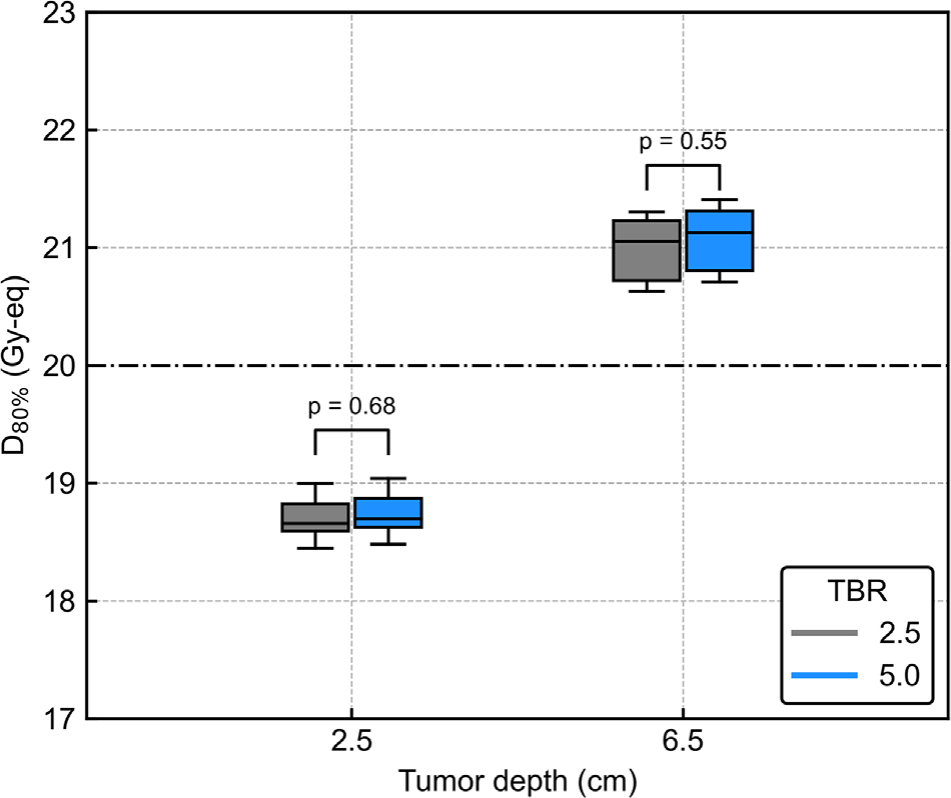
Box plots of *D*_80%_ for the 3 cm lateral shift with different T/B ratios. *P*‐values in the graphs are evaluated by the Student's *t*‐test

Figure 12 shows *D*_80%_ as a function of the AP‐shift distance with different T/B ratios. The ANOVA results showed no significant differences in the DIs against the AP shift between the different T/B ratios for both *T*_2.5_ and *T*_6.5_ (*p* = 0.88 and 0.81). Figure 13 shows the box plots of *D*_80%_ for the 3‐cm lateral shift with different T/B ratios. The Student's *t*‐test showed no significant differences in DIs between different T/B ratios for both *T*_2.5_ and *T*_6.5_ (*p* = 0.68 and 0.55). The DVHs of the AP‐shifted and lateral‐shifted plans with different T/B ratios are also summarized in Figures S7–8.

### 3.5 Effect of ^10^B concentration

**FIGURE 14:**
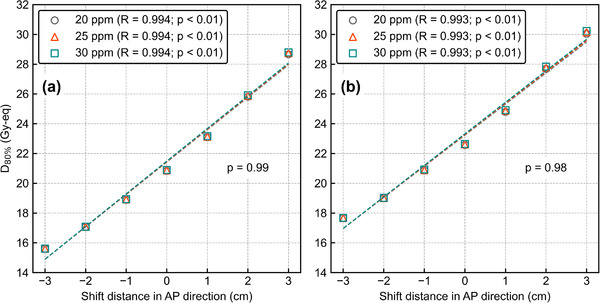
*D*_80%_ as a function of the shift distance in the anterior–posterior direction with different ^10^B concentrations for (a) *T*_2.5_ and (b) *T*_6.5_. The *R* values in the legend represent the correlation coefficients. All data have a high correlation between the anterior–posterior shift and *D*_80%_ and *p*‐values less than 0.01. Other *p*‐values in the graphs are evaluated by two‐way ANOVA

**FIGURE 15:**
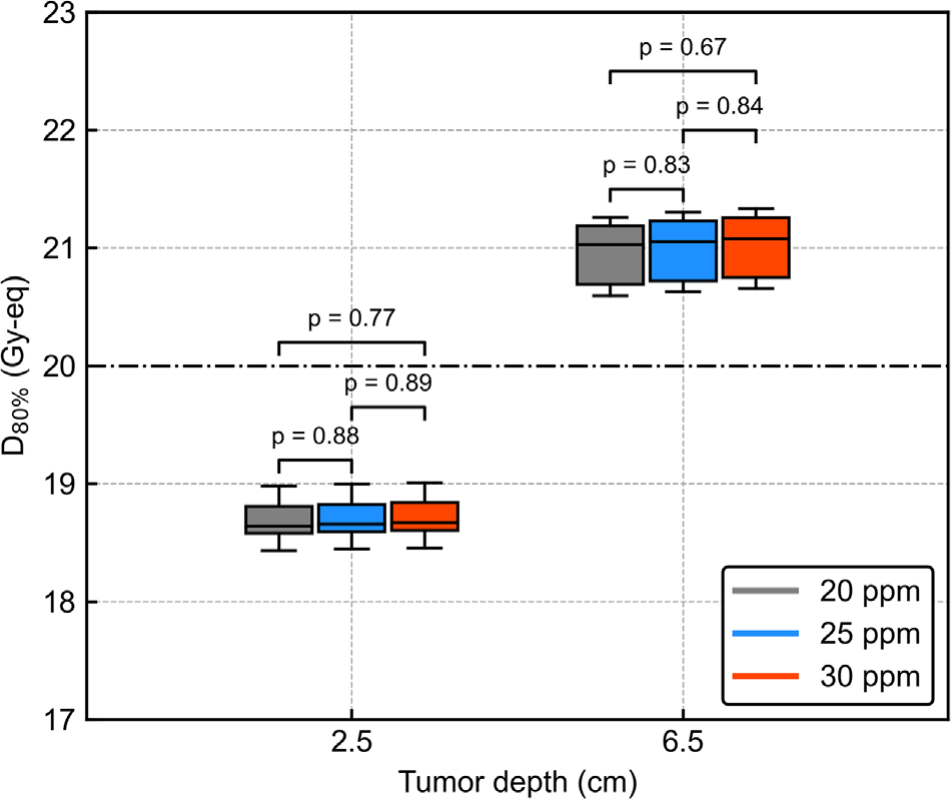
Box plots of *D*_80%_ for the 3 cm lateral shift with different ^10^B concentrations. *P*‐values in the graphs are evaluated by the Student's *t*‐test

Figure 14 shows *D*_80%_ as a function of the AP‐shift distance with different ^10^B concentrations. The ANOVA showed no significant difference in the DIs against the AP shift between the three ^10^B concentrations (*p* = 0.99 and 0.98). Figure 15 shows the box plots of *D*_80%_ for the 3‐cm lateral shift with different ^10^B concentrations. The Student's *t*‐test showed no significant differences in DIs between all ^10^B concentrations (*p* > 0.05 for all comparisons). The DVHs of the AP‐shifted and lateral‐shifted plans with different ^10^B concentrations are also summarized in Figures S9–10.

## 4 DISCUSSION

Understanding the DI against position errors in BNCT is as important as in other radiation therapy modalities. From the above results, the coefficients of MRA in the AP shift are 2.16 and 2.08 for *T*_2.5_ and *T*_6.5_, whereas those of all lateral shifts are less than 0.2. Thus, the DIs were found to mainly depend on the AP‐directional shift rather than the lateral shift, which is supported by the MRA. Additionally, subgroup analyses of the DIs in terms of the collimator size, CSD, T/B ratio, and ^10^B concentration were performed. Significant differences in the DIs against the AP shift evaluated by two‐way ANOVA were observed under different collimator sizes for *T*_2.5_ and under different CSDs for *T*_6.5_ (*p* < 0.05). Significant differences in DIs against the lateral shift evaluated by the Student's *t*‐test were observed for different collimator sizes and different CSDs for *T*_2.5_ (*p* < 0.05). The results indicate that an inaccurate patient positioning in AB‐BNCT may lead to significant dose errors, especially in the AP shift, and may be affected by collimator size and CSD. So, a method to improve the robustness of patient positioning may be necessary.

Lee et al. evaluated DIs against position errors using a cylindrical and human‐head‐modeled phantom in reactor‐based BNCT in Taiwan.^10^ The results indicated the DIs in the AP‐directional shift were greater than those in the lateral shift. These results are consistent with ours. Because both sets of DIs between the reactor and the accelerator generate low‐energy neutrons enough to be scattered in the air and given the larger air gap due to the AP shift steeply reduces the thermal neutron flux, both sets must exhibit similar tendencies. However, uniform treatment parameter settings of the collimator size, CSD, T/B ratio, and ^10^B concentration were employed in the Lee et al. study that is a generous assumption considering that the T/B ratio and ^10^B concentration are unique among patients who undergo BNCT.^17^, ^18^ Collimator sizes are selected depending on the patient anatomy (location of tumor and risk organs) in clinical BNCT,^9^ and the CSD varies depending on the tumor location and patient positioning. It is prudent to investigate DIs under various parameter settings. To the best of our knowledge, no study on DI against patient position errors in AB‐BNCT has focused on the treatment parameter settings, as stated above. This study would be useful for creating a robust BNCT treatment plan considering patient setup errors or intrafractional patient motion. For example, to suppress the tumor dose reduction due to position error, a larger collimator size and larger CSD are preferable. However, a larger collimator size leads to a higher dose to the surrounding normal tissues, and larger CSD extends the treatment time. The treatment planner of BNCT must understand the risk‐benefit ratio and select the optimal treatment plan.

The CVs were greater for larger shift distances in the DI evaluation of the overall shifts as expected. Those of *T*_2.5_ were greater than those of *T*_6.5_. As shown in Figure 5, the boron dose distribution is steeper near the surface. That is, these differences in the slope of the dose distribution between *T*_2.5_ and *T*_6.5_ might generate differences in DI variation against the position error because the boron dose is mainly related to the total RBE‐weighted dose in the tumor. The HI values were 0.16, 0.18, and 0.20 for *T*_2.5_ and 0.41, 0.46, and 0.51 for *T*_6.5_ in the shift distance of 1.0, 2.0, and 3.0 cm. The HI values were higher for deeper tumor locations. A higher HI value indicates a lower uniformity of the dose distribution as per the definition. As shown in Figure S1, *T*_6.5_ is located at the point where the thermal neutron flux is drastically decreasing, whereas *T*_2.5_ is located at the peak of the flux. Therefore, the RBE‐weighted dose in *T*_6.5_ decreased drastically at deeper points, and the HI value increased. The neutron beam irradiated from the AB‐neutron source has low directivity and is diffused, unlike photons or other particles. Due to this characteristic, more neutrons escape in the lateral direction under the condition of greater CSD, and AP‐directional shift might generate a greater reduction in the RBE‐weighted dose than the lateral shift.

The DIs against the AP shift were significantly smaller under larger collimator sizes for *T*_2.5_, as shown in Figures 6 and 7. Meanwhile, the DIs against lateral shift were significantly smaller under larger collimator sizes for both *T*_2.5_ and *T*_6.5_, and were more pronounced for *T*_2.5_, as shown in Figures 8 and 9. Hu et al. measured the thermal neutron flux on‐ and off‐axis from the NeuCure® system and compared it with its TPS, the data of which are summarized in Figures S1–2. As shown in Figure S1, the peak position of the percentage depth thermal neutron flux is slightly deeper for larger collimator sizes. Meanwhile, a greater difference in the off‐axis ratio of the thermal neutron flux at a depth of 2 cm can be observed between the collimator sizes than that at a depth of 6 cm, as shown in Figure S2. This effect might result in smaller DIs for *T*_6.5_. The DIs against the AP shift were smaller under larger CSD and significant for *T*_6.5_, as shown in Figure 10. As shown in Figure S6, the slope of the boron dose falloff tapers off at deeper locations. Therefore, a greater CSD results in smaller DIs for position errors. The effect of the T/B ratio or ^10^B concentration on the DIs against the position error might be small because the ^10^B concentration in the tumor is too low (less than a few hundred ppm) to affect the thermal neutron flux distribution.

This study has several limitations. First, a real patient dataset was not used. The patient setup for BNCT varies depending on the tumor location and positioning. In addition, for clinical BNCT, the dose is prescribed to the organ at risk. Therefore, it was preliminarily desirable to use more general and nonpatient‐specific tendencies of DIs against position errors using a simple phantom model. In future work, we will investigate the DIs for each patient. Second, although the patient shift distance was assumed to be 1.0, 2.0, or, 3.0 cm, the actual setup error or intrafractional patient motion is uncertain. The actual shift might be small because patients are firmly held using a thermoplastic mask commonly used for X‐ray therapy. Currently, the intrafractional patient motion is monitored using a video camera where the images are viewed from outside of the irradiation room. In our future work, we will monitor intrafractional patient motion using motion capture devices, such as infrared reflective markers, to evaluate the distribution of the actual delivered dose based on the monitored results. Third, only an RBE‐weighted dose was applied for the dose evaluation. Another formalism “photon iso‐effective dose” is proposed as RBE‐weighted dose overestimates the dose delivered at higher dose regions.^19^ Although it is desirable to additionally evaluate the DI using the photon iso‐effective formalism, the NeuCure Dose Engine® only applies the conventional RBE‐weighted dose and the setting cannot be changed from the user's side. Fourth, it is uncertain how these DIs can influence the clinical outcome of the treatment. The dose of current BNCT is prescribed for normal tissues such as mucosa and skin. The decrease of tumor control probability due to the position errors might be negligible when the tumor dose is extremely high. However, the tumor control probability might be decreased when the tumor dose is low due to a deeper location or being close to the organs at risk. It is important to mitigate the risks from a physical or technical perspective. This study may enable more appropriate treatment plans and may lead to improvement of tumor control probability.

## 5 CONCLUSIONS

A comprehensive evaluation of DIs against patient position errors in AB‐BNCT using a phantom model was performed. Consistent with a previous similar reactor‐based BNCT study by Lee et al., the DIs were found to mainly depend on the AP‐directional shift. Hence, patient shift away from the collimator may greatly reduce the tumor dose. When setting up the patients, it is necessary to be particularly rigorous to avoid patient motion in the beam axis direction. Significant differences in the DIs were observed under different collimator sizes and CSD, but not between the T/B ratio and ^10^B concentration in the blood. Our results indicate that a larger collimator size and a larger CSD may lead to robustness of tumor dose, although this action should be performed considering patient characteristics such as patient anatomy and tumor location. This study may help improve the robustness of BNCT planning against patient shifts.

## CONFLICT OF INTERESTS

The authors have no conflict to disclose.

## Supporting information


**Figure S1**: Percentage depth thermal neutron flux with different collimator size referred from Hu et al [1]
**Figure S2**: Off‐axis ratio of thermal neutron flux at from water phantom surface with different collimator size referred from Hu et al. [1].
**Figure S3**: DVHs of 3‐cm lateral shift with different collimator size for *T*6.5
**Figure S4**: DVHs of different shift distance in AP direction and CSD for (a) *T*2.5 and (b) *T*6.5
**Figure S5**: DVHs of lateral shift direction with different CSD for (a) *T*2.5 and (b) *T*6.5**Figure S6**: Boron dose falloff as a function of CSD
**Figure S7**: DVHs of different shift distance in AP direction and T/B ratio for (a) *T*2.5 and (b) *T*6.5
**Figure S8**: DVHs of lateral shift direction with different T/B ratio for (a) *T*2.5 and (b) *T*6.5
**Figure S9**: DVHs of different shift distance in AP direction and ^10^B concentration for (a) *T*2.5 and (b) *T*6.5
**Figure S10**: DVHs of lateral shift direction with different ^10^B concentration for (a) *T*2.5 and (b) *T*6.5

## A.1 BNCT dose component

The concept of total dose was introduced in IAEA‐TECDOC‐1223.^20^ Recently, Kumada et al. summarized a review on BNCT treatment planning and proposed a more detailed dose calculation.^8^ The RBE‐weighted dose *D_T_* (Gy‐eq) for BNCT^8^ can be evaluated using the following equation:

(A.1)
$$D_{n}=\int_{t}\int_{E}f_{n}\left(E\right)\;\varphi \left(E,\;t\right)dEdt,$$

(A.2)
$$\begin{array}{ccc} D_{T} & = & CBE\times D_{B}+RBE_{H}\times D_{H}+RBE_{N}\times D_{N}\\ & & +RBE_{\gamma }\times D_{\gamma ,} \end{array}$$

where *D_n_* is the absorbed dose arising from reactions between neutrons and atoms, *f* is a factor for the kinetic energy released in the matter (KERMA) for neutrons or the dose conversion factor of photons, *φ*(*t*) is the flux of neutrons or photons at a given point, *D_B_* is the dose due to the ^10^B(n, α)^7^Li reaction, *D_P_* is that due to the ^1^H(n, n)p reaction, *D_N_* is that due to the ^14^N(n, p)^14^C reaction, and *D_γ_* is the gamma‐ray dose emitted from the neutron beam port and induced in the tissue due to the ^1^H(n, γ)^2^H reaction. *CBE* is the compound biological effectiveness value. *RBE_H_* and *RBE_N_* are the RBE for hydrogen and nitrogen, respectively Table A.1.

**TABLE A.1:** CBE and RBE parameters used for dose calculation

	CBE	RBE_N_	RBE_H_	RBE_γ_
Tumor	3.80^21^	2.90	2.40	1.00
Tissue soft	1.35^22^	2.90	2.40	1.00
