# Supplementary material for: Comprehensive evaluation of dosimetric impact against position errors in accelerator‐based BNCT under different treatment parameter settings
Source: Med Phys. 2022 Jul 4;49(8):4944–54. doi: 10.1002/mp.15823 (PMC9541895; doi:10.1002/mp.15823)
Supplement: Supplementary file 1 — Figure S1: Percentage depth thermal neutron flux with different collimator size referred from Hu et al [1] Figure S2: Off‐axis ratio of thermal neutron flux at from water phantom surface with different collimator size referred from Hu et al. [1]. Figure S3: DVHs of 3‐cm lateral shift with different collimator size for T6.5 Figure S4: DVHs of different shift distance in AP direction and CSD for (a) T2.5 and (b) T6.5 Figure S5: DVHs of lateral shift direction with different CSD for (a) T2.5 and (b) T6.5Figure S6: Boron dose falloff as a function of CSD Figure S7: DVHs of different shift distance in AP direction and T/B ratio for (a) T2.5 and (b) T6.5 Figure S8: DVHs of lateral shift direction with different T/B ratio for (a) T2.5 and (b) T6.5 Figure S9: DVHs of different shift distance in AP direction and 10B concentration for (a) T2.5 and (b) T6.5 Figure S10: DVHs of lateral shift direction with different 10B concentration for (a) T2.5 and (b) T6.5 [file MP-49-4944-s001.pdf]

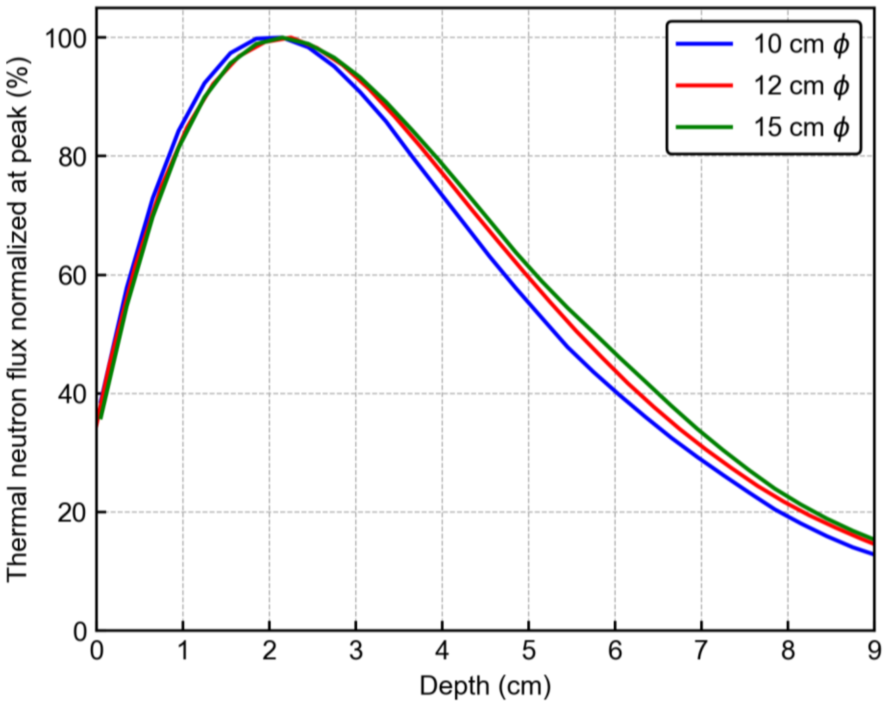

Figure S1: Percentage depth thermal neutron flux with different collimator size referred from Hu et al [1].

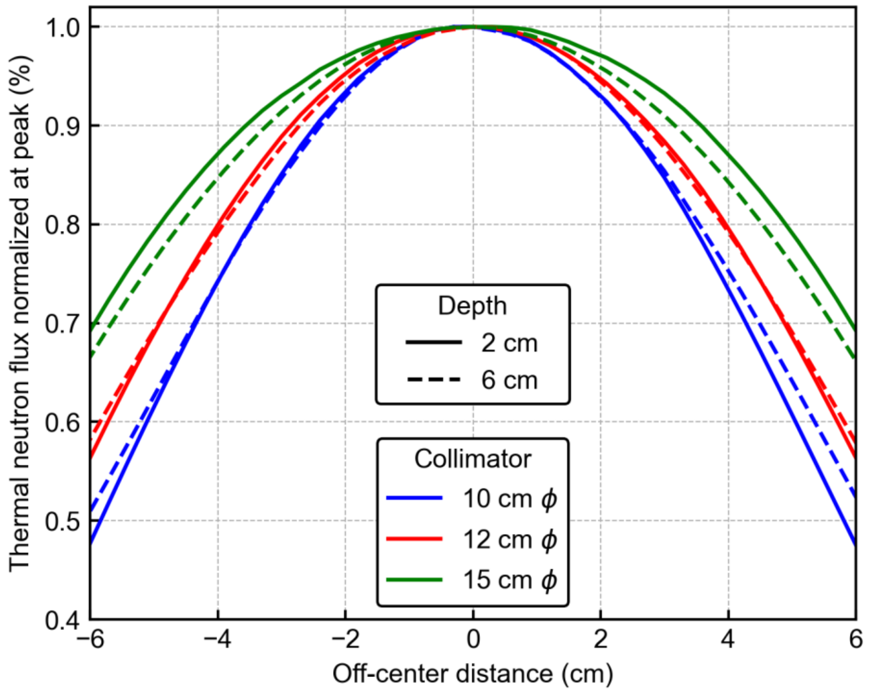

Figure S2: Off-axis ratio of thermal neutron flux at from water phantom surface with different collimator size referred from Hu et al [1].

1. Hu, N., et al., Radiat Oncol, 2021. 16(1): p. 243.

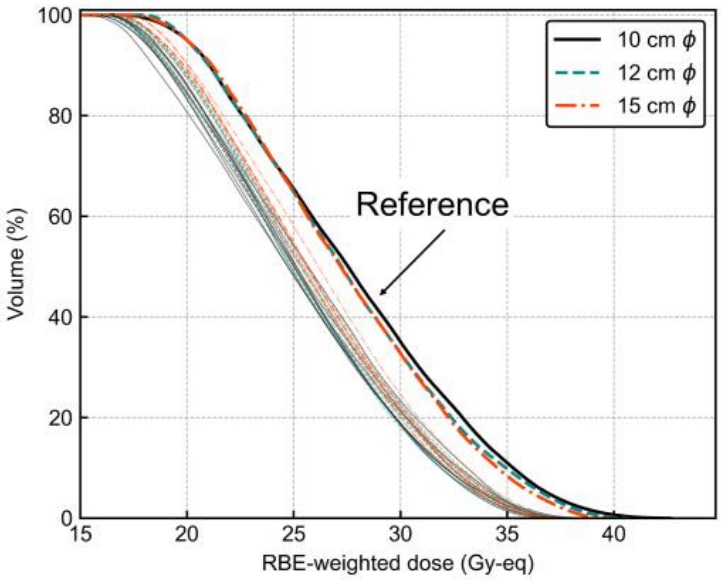

Figure S3: DVHs of 3-cm lateral shift with different collimator size for $T_{6.5}$.

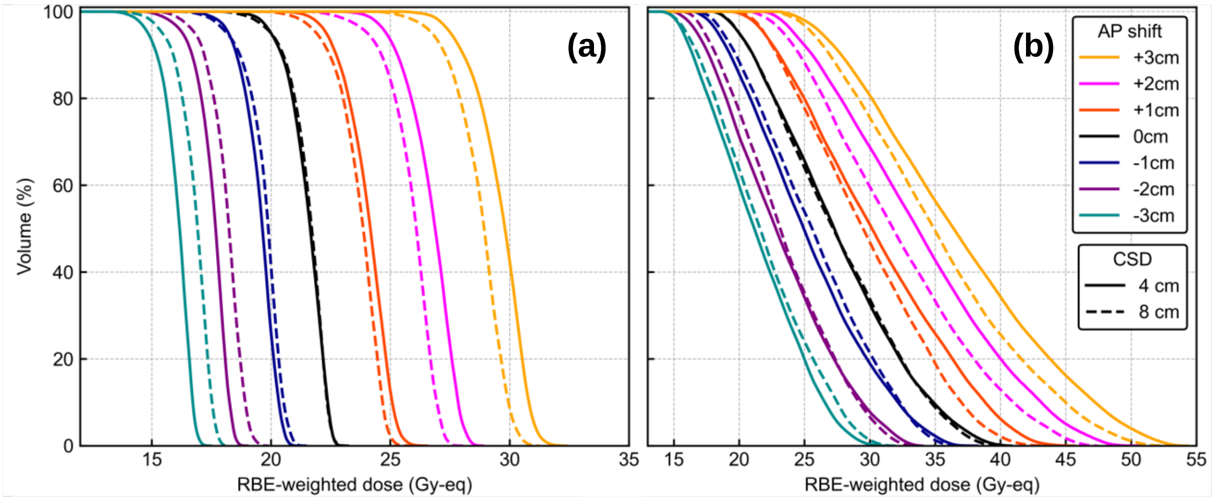

Figure S4: DVHs of different shift distance in AP direction and CSD for (a) $T_{2.5}$ and (b) $T_{6.5}$.

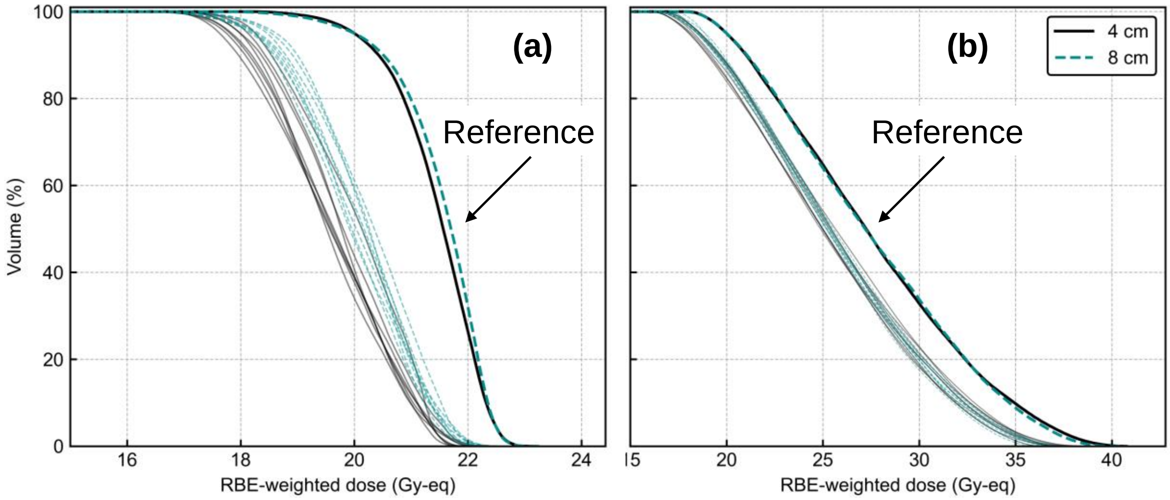

Figure S5: DVHs of lateral shift direction with different CSD for (a) $T_{2.5}$ and (b) $T_{6.5}$.

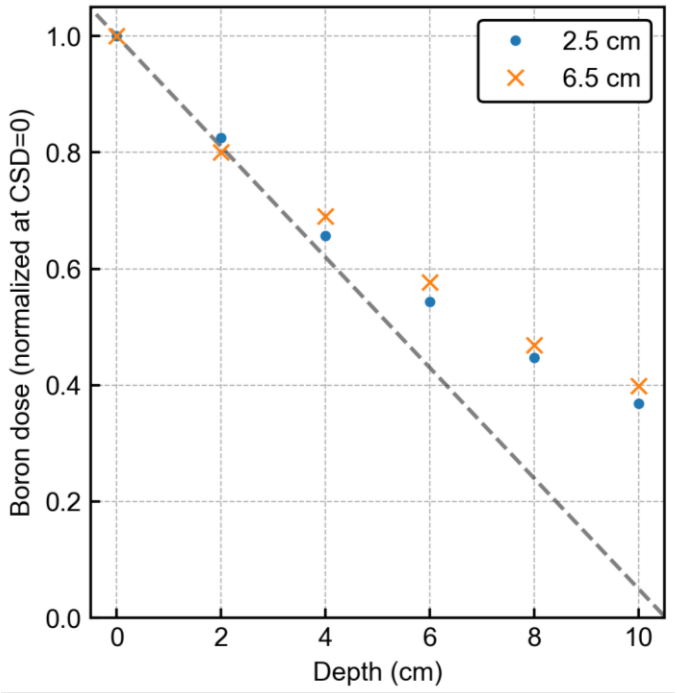

Figure S6: Boron dose fall-off as a function of CSD. The black line shows an assumption of dose fall-off in linearity. The slope of dose fall-off becomes gentler in deeper location.

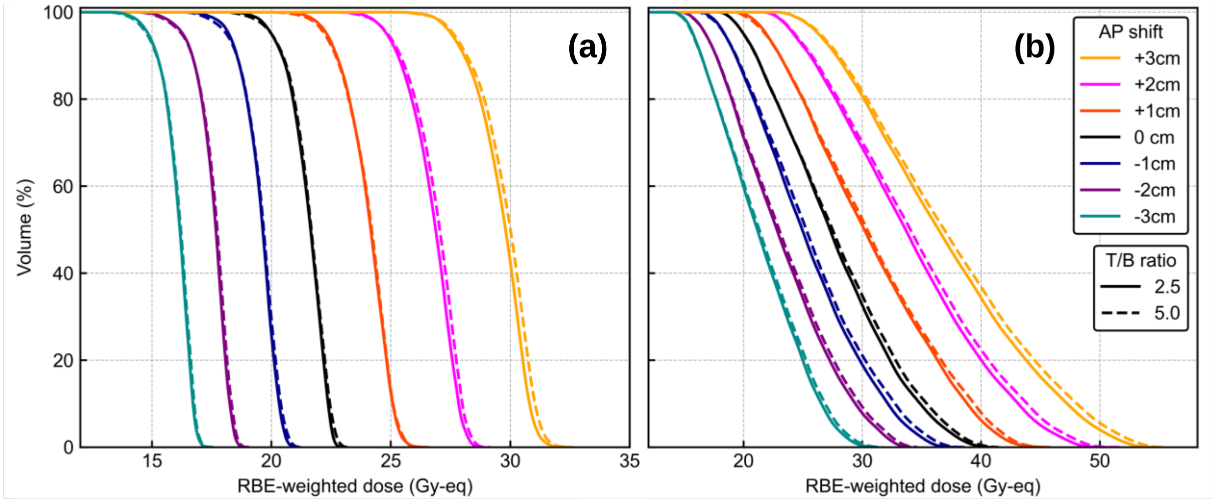

Figure S7: DVHs of different shift distance in AP direction and T/B ratio for (a) $T_{2.5}$ and (b) $T_{6.5}$.

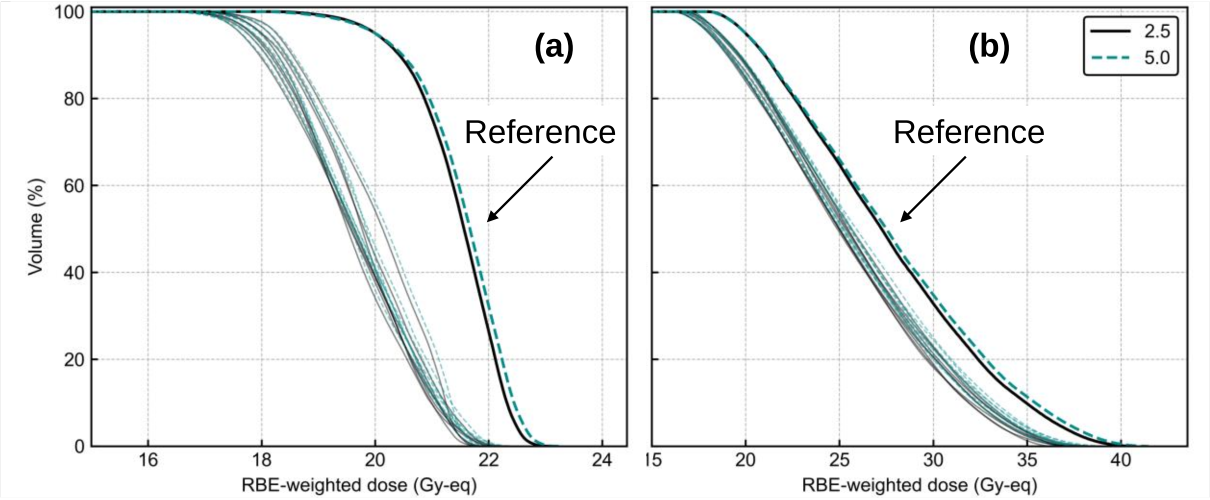

Figure S8: DVHs of lateral shift direction with different T/B ratio for (a) $T_{2.5}$ and (b) $T_{6.5}$.

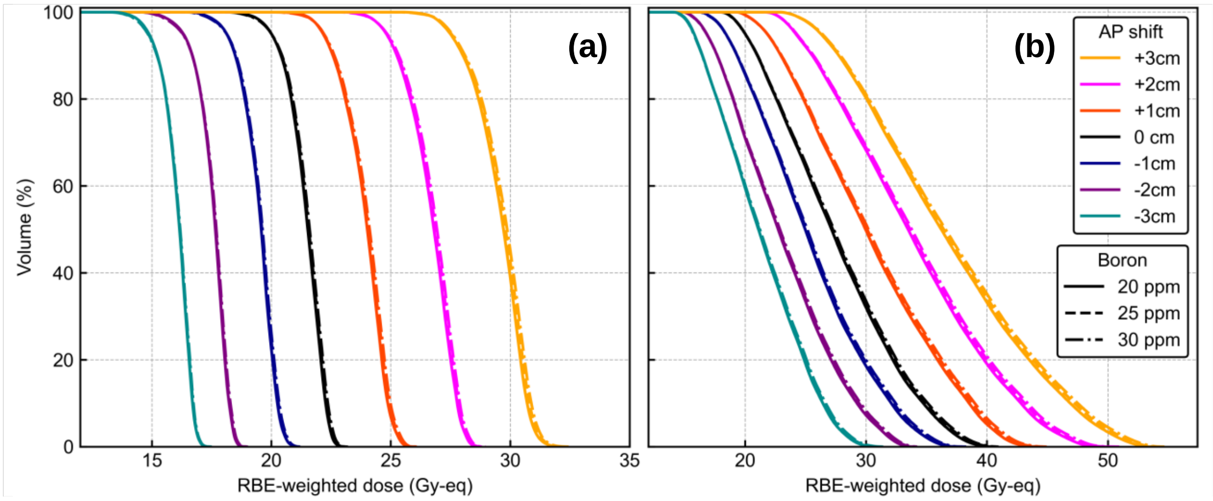

Figure S9: DVHs of different shift distance in AP direction and ^{10}B concentration for (a) $T_{2.5}$ and (b) $T_{6.5}$.

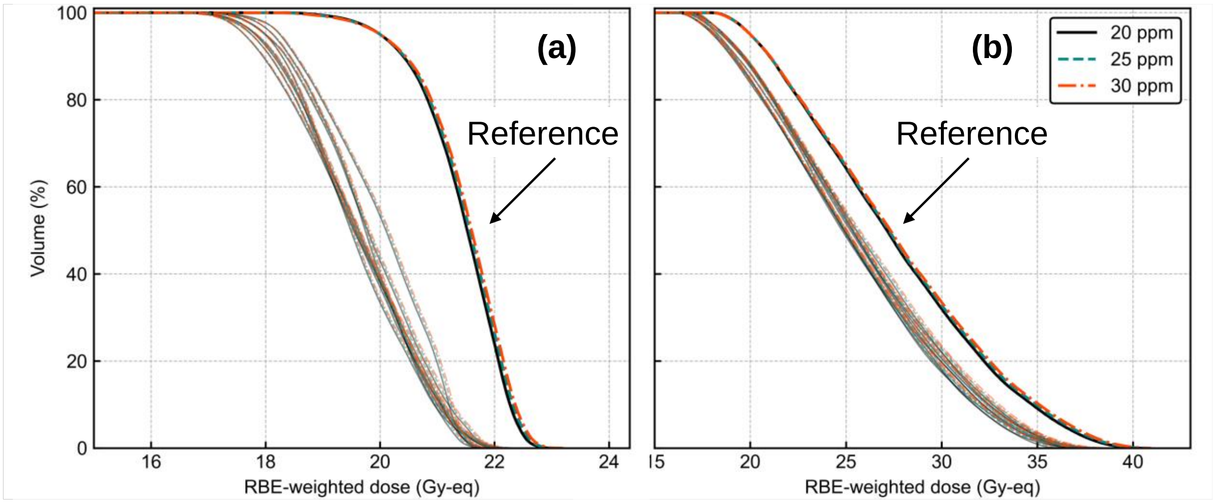

Figure S10: DVHs of lateral shift direction with different ^{10}B concentration for (a) $T_{2.5}$ and (b) $T_{6.5}$.
